# Current landscape of research ethics consultation services: National survey results

**DOI:** 10.1017/cts.2022.470

**Published:** 2022-11-08

**Authors:** Holly A. Taylor, Kathryn M. Porter, Connor Sullivan, Jennifer B. McCormick

**Affiliations:** 1 Department of Bioethics, Clinical Center, National Institutes of Health, Bethesda, MD, USA; 2 Treuman Katz Center for Pediatric Bioethics, Seattle Children’s Research Institute, Seattle, WA, USA; 3 Department of Humanities, College of Medicine, Pennsylvania State University, Hershey, PA, USA

**Keywords:** Research ethics, consultation, human subject research, national survey, competencies

## Abstract

**Introduction:**

The goal of a research ethics consultation service (RECS) is to assist relevant parties in navigating the ethical issues they encounter in conduct of research. The goal of this survey was to describe the current landscape of research ethics consultation and document if and how it has changed over the last decade.

**Methods:**

The survey instrument was based on the survey previously circulated. We included a number of survey domains from the previous survey with the goal of direct comparison of outcomes. The survey was sent to 57 RECS in the USA and Canada.

**Results:**

Forty-nine surveys were completed for an overall response rate of 86%. With the passing of 10 years, the volume of consults received by RECS surveyed has increased. The number of consults received by a subset of RECS remains low. RECS continues to receive requests for consults from a wide range of stakeholders. About a quarter of RECS surveyed actively evaluate their services, primarily through satisfaction surveys routinely shared with requestors. The number of RECS evaluating their services has increased. We identified a group of eight key competencies respondents find as key to providing RECS.

**Conclusions:**

The findings from our survey demonstrate that there have been growth and development of RECS since 2010. Further developing evaluation and competency guidelines will help existing RECS continue to grow and facilitate newly established RECS maturation. Both will allow RECS personnel to better serve their institutions and add value to the research conducted.

## Introduction/Background

The goal of a research ethics consultation service (RECS) is to assist relevant parties in navigating the ethical issues they encounter in the conduct of research. Their function is to “provide information, analyze, and deliberate about ethical issues, and recommend a course of action” [1]. A recent publication articulates the value RECS can add specifically to the promotion of ethical human participant research in at least four contexts: “(1) as a resource for investigators before and after regulatory review; (2) as an additional resource for investigators, Institutional Review Boards (IRBs), and other research administrators facing challenging and novel ethical issues; (3) to assist IRBs and investigators with the increasing challenges of informed consent and risks/benefit analyses; and (4) as a flexible resource that can provide collaborative assistance to overcome study hurdles, mediate conflicts within a study team, or even directly engage with research participants” [2].

RECS were first established in the late 1980s and early 1990s [3,4]. These early RECS were often created to meet an institutional need, either to support an institutional effort in a challenging area of biomedical science or to enhance educational efforts by providing investigators with study-specific advice [5,6]. There was a swell of interest in RECS in 2005 when a number of institutions funded during the first rounds of the National Center for Advancing Translational Science (NCATS; previously National Center for Research Resources) Clinical and Translational Science Awards (CTSA) proposed to establish a RECS to convey to the NCATS an institutional commitment to ethics [3,4].

Thanks to an NCATS initiative to foster collaboration across CTSAs, those running RECS across the country met and began to collaborate. By 2010, McCormick and her colleagues concluded it was time to conduct a national survey to document this emerging research resource so that their composition and function could be determined [3]. They found that 70% of the CTSAs funded at the time had a RECS (33 of 46) and of those, about half had completed at least one consult.

An important component of NCATS commitment to clinical and translational research capacity was the creation of a number of Key Function Committees (KFC) to coordinate activities across funded sites, including a KFC on Clinical Research Ethics (KFC-CRE). A subcommittee (the Consultation Working Group) of the KFC-CRE consisted of individuals providing, or planning to provide, RECS at their respective institutions. When the CTSA KFCs were disbanded in 2014, the Consultation Working Group decided to continue their work together and became the Clinical Research Ethics Consultation Collaborative (CRECC) [7]. The publication of the 2010 survey results became the first of a number of publications authored by small groups of CRECC members [2,8,9,10].

Because each RECS is unique to its home institution, each has its own scope of services, policies, and practices. Consultants have addressed RECS-related policy issues and made efforts to standardize the consultation process. For example, Lavery et al. identified strategies that RECS consultants can apply to consultations concerning investigators from high-income countries that are conducting research in low- and middle-income countries [11]. Sharp et al. identified the intersecting responsibilities of RECS members, their exposure to sensitive information, and difficulties describing the consultation process to requestors as challenges faced by consultation services [9]. Cho and colleagues created a standardized data collection tool and repository for consultations [8]. The tool was then adopted by 11 RECS and the findings reported [2].

More recently a number of publications have hinted at the continued growth and development of RECS [2,10]. The goal of this survey was to describe the current landscape of research ethics consultation and document if and how it has changed over the last decade. As the field has had 10 years to mature, the survey was also designed to elicit opinions about how best to evaluate the quality of RECs and determine which professional competencies those delivering RECs consider the most important for current and future consultants to pursue.

## Materials and Methods

### Survey

The survey instrument was based on the survey previously circulated [3]. We included a number of survey domains from the previous survey with the goal of comparison. These domains included: consultant staffing, funding, access and utilization, reporting, tracking and evaluation, and relationships with IRB. In order to keep the approximate length of the survey similar, we dropped questions regarding policies related to privacy and confidentiality and added a question about the most common type of issue brought to the consult service, expanded the number of questions in the evaluation domain and added a section to elicit opinions about key professional competencies related to the delivery of RECS. On the latter, two of the authors (HAT and KMP) were involved in a sub-committee of CRECC members and members of the American Society for Bioethics and Humanities Clinical Research Ethics Consultation Affinity Group that reviewed and considered the relevant literature on the development of competencies for the clinical ethics consultation setting [10]. Based on this review, the current clinical ethics consultation competencies themselves, and the collective knowledge of the committee, the group developed a set of research ethics consultation competencies [10,12]. These competencies were included in the survey to collect feedback from the broader RECS community (see Table 1). Between 2012 and 2013 10 RECS, making up the CRECC Repository Group, contributed key consult data in order to look for trends across sites [2]. Informed consent was by far the most common concern addressed across consults. The next most frequent topics were research/clinical practice relationships; benefit risk assessment; participant selection/recruitment; disclosure of incidental findings; privacy/confidentiality; and study design [2]. These common topics were included in the survey for comparison.

**Table 1.** Competencies (total n = 42)

**Knowledge: Fundamental ethical principles related to research with humans subjects (64%), Research regulations (54%), Basic study design (50%),** Familiarity with dominant ethical theories and ethical concepts that typically emerge in the research setting (43%), Frameworks for ethical analysis (43%), How to obtain effective consent (including concepts such as competence, capacity and undue influence) (24%), Conflicts of interest policy (3%), Privacy requirements (0%)
**Assessment: Identify and analyze the ethical dimensions and value conflicts related to the request (69%),** Differentiate research ethics questions from those regarding clinical ethics, regulatory concerns, culture/customs/norms, personal opinion (24%), Access relevant literature, policies, and standards (5%)
**Process: Conduct ethical analyses or moral reasoning (76%), Gather relevant data (67%), Facilitate meetings and conduct deliberations (62%),** understand the organization (21%), leverage resources (16%), develop and communicate the RECS mission and expectations (14%), document cases and other RECS activities (5%)- implement quality improvement (5%), identify systems (2%)
**Interpersonal: Elicit relevant information to obtain full picture of the ethical concerns (32, 76%)**- Communicate across disciplines (43%), Listen (24%), Facilitate deliberation (19%), Maintain openness to multiple perspectives (17%), Lead productive conflict resolution (10%), Attend to various relational barriers to communication (5%)

The survey was pilot tested with a group of colleagues familiar with RECS but not active consultants themselves. The survey was uploaded into Survey Monkey (https://www.surveymonkey.com) for dissemination.

### Sampling Frame

**Fig. 1.**
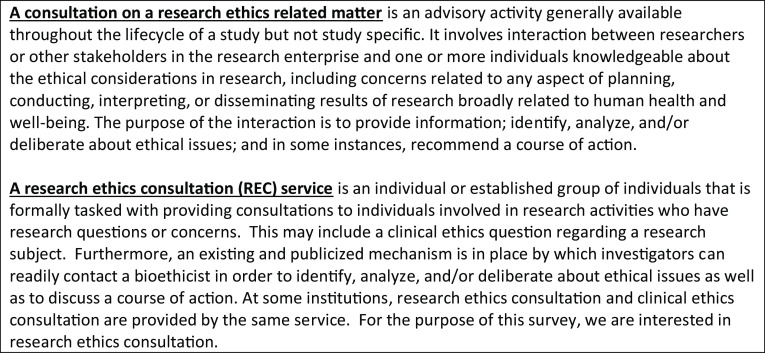
Research Ethics Consultation Service.

Prior to survey distribution, we sought to create an exhaustive sampling frame. Many current CRECC members lead or have a role in RECS across the USA and a few in Canada. Our sampling frame started with CRECC members by asking them to confirm the primary contact for their RECS and refer us to RECS they were aware of that were not represented in the CRECC. In addition, we asked the President of the Association of Bioethics Program Directors to send an email on our behalf asking its members whether their institution has a RECS and if so, to provide contact information. We also sent a similar request to a listserv used to connect CTSA Administrators. Finally, we did a Google search on keywords relevant to RECS. In an effort to expand the number of Canadian RECS in our sample, we reached out to the Secretariat on Responsible Conduct of Research at the Canadian Institute for Health Research and the Canadian Association of Research Ethics Boards but were not successful in identifying any additional stand-alone RECS. In Canada, RECS are most commonly embedded within the Human Subject Protection Programs, making the Canada RECS different from US RECS in form and function. As such we hope a future project will explore the delivery of research ethics consultations in Canada. Based on these methods, we identified a sampling frame of 53 RECS in the USA and 4 in Canada for a total of 57. Because we were interested in the characteristics of the RECS rather than details about individuals, respondents were asked to complete the survey on behalf of their RECS. Only respondents affiliated with a formal consult service (See Fig. 1 for definitions) completed the survey.

### Data Collection

We sent a link to the survey to all 57 contacts in July 2021. The survey included a disclosure statement noting the key elements of consent including the fact that participation was voluntary. We asked each respondent to provide their name and their institution to make sure we did not receive two completed surveys from the same institution. This information was stripped from surveys by one author (HAT) after the survey was submitted and stored separately from the survey data. Those who did not complete the survey received up to three reminders. The project was reviewed and determined to not be human subjects research by the [IRB name withheld for review] and as exempt from IRB review by [names of two other IRBs withheld for review].

### Analysis

Univariate analysis of close-ended survey questions was conducted with tools embedded in SurveyMonkey and included in Data Tables. Proportions between groups were compared using a Pearson Chi-square test. An exact version of this test was used if small cell counts made the asymptotic version invalid. Proportional comparisons were performed using SAS version 9.4 (SAS Institute, Cary, NC). Open-ended responses were reviewed and sorted into themes.

## Results

A total of 49 surveys were completed for an overall response rate of 86%. Of the US RECS, 46 out of the 53 (86%) responded while 3 of the 4 (75%) Canadian RECS responded. Six respondents (all from US institutions) indicated they did not *currently* have a formal RECS. The findings below include only those respondents representing a formal RECS and therefore are based on a total sample of 43.

The difference between the number of existing RECS in 2010 and in 2021 is statistically significant (see Table 2). Close to two-thirds of RECS surveyed in 2021 have been in existence for 6 or more years and close to two-thirds of those for more than 10 years (see Table 3). In 2010, only two services had been in existence for more than 6 years [3]. This difference between 2010 and the current survey is statistically significant (see Table 3)

**Table 2.** Key proportional comparisons

Question	2010 proportion	2021 proportion	*p*-value
Number who responded yes to having a service.	33/46 (71.7%)	43/49 (87.8%)	0.051
Number who indicated they were conducting an evaluation of any kind.	5/33 (15.2%)	12/42 (28.6%)	0.168

**Table 3.** Basic demographics with key comparisons

Demographic	2010 *n* = 35 n/total (%)	2021 *n* = 43 n/total (%)	*p*-value
**RECS establishment**			
<1 year	6/21 (28.6%)	3/43 (7.0%)	0.027
1–2 years	5/21 (23.8%)	2 /43 (4.7%)	0.032
3–5 years	8/21 (38.1%)	5/43 (11.6%)	0.020
6+ years	2/21 (9.5%)	27/43 (62.8%)	< 0.001
Not sure	0/21 (0.0%)	6/43 (14.0%)	0.167
**Number of core consultants**			
1–2	10/33 (30.3%)	20/43 (47.6%)	0.129
3–5	19/33 (57.6%)	11/43 (26.2%)	0.006
6–8	4/33 (12.1%)	7/43 (16.7%)	0.742
Still being determined	0/15 (0.0%)	3/43 (7.1%)	0.249
Not sure	0/15 (0.0%)	1/43 (2.4%)	1.0
**Number of consults in past year**			
None	0/15 (0.0%)	4/43 (9.3%)	0.346
1–4	8/15 (53.3%)	14/43 (32.6%)	0.220
5–10	1/15 (6.7%)	9/43 (20.9%)	0.268
11–15	3/15 (20.0%)	5/43 (11.6%)	0.675
16–25	2/15 (13.3%)	5/43 (11.6%)	1.0
26 or more	1/15 (6.7%)	3/43 (7.0%)	1.0
Not sure	0/15 (0.0%)	3/43 (7.0%)	0.564

*Frequencies for any given item will not necessarily total 43 because some questions allowed multiple responses and all participants did not respond to all questions.

RECS = research ethics consultation service

### Affiliation with Bioethics Centers

More than three-quarters of RECS survey indicated their home institution has a Center, Department or Program in Bioethics. More than half of those RECS at an institution with a Bioethics center indicated that their service is a component of their Bioethics center (rather than independent of the center).

### Number and Most Common Type of Consults

**Fig. 2.**
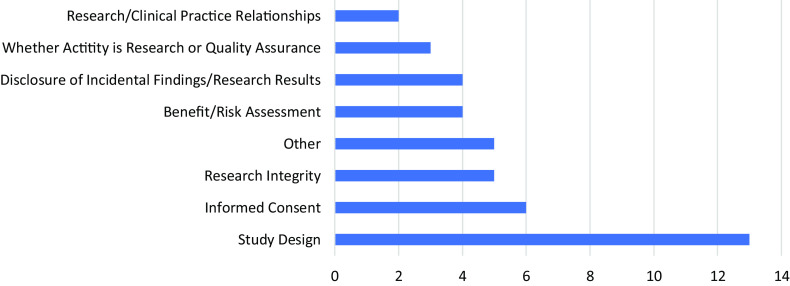
Most common type of ethical concern brought to research ethics consultation services (*n* = 42).

Only four of the current respondents had not completed a single consult in the prior year. In 2010, more than half of the RECs surveyed had not completed a single consult in the prior year [3]. The same proportion of current and past respondents had completed 15 or fewer consults in the past year [3]. The difference in “number of consults in the past year” between 2010 and 2021 is not significant across all categories (see Table 3). When asked about the content of the total consults completed to date, the RECS surveyed reported that study design was the most common type of ethical concern brought to the RECS followed by informed consent (see Fig. 2). 2010 respondents were not asked to identify the common ethical concerns brought to the RECS [3].

*Type of Consultee:* Almost all of the RECS surveyed accept consults from Principal investigators (PIs), study team members, Offices of Research Administration, Institutional Review Boards (IRBs)/Research Ethics Boards (REB) staff and members, clinicians, and trainees. The same was true of the respondents in 2010. Similar proportions (50%) of respondents in 2010 and 2021 accept consults from study participants and/or their family/caregiver and about one-third of the RECS surveyed (30%) provide consults to individuals/groups unaffiliated with their institution (e.g. industry). In response to a follow-up question about the most frequent requestors of consults, PIs were identified as the most frequent users (60%), followed by study team members (20%). Respondents in 2010 also identified these groups as the most common requesters [3].

*Core Consultants:* Almost half of the RECS surveyed are staffed by 1–2 individuals while a quarter are staffed by 3–5 individuals. These proportions were reversed in 2010 with more than half served by 3–5 and one-third staffed by 1–2 [3]. The difference between 2010 and 2021 for the proportion of RECS with 3–5 core consultants is statistically significant (see Table 3). Almost all RECS surveyed are staffed by at least one consultant with an academic background in bioethics or applied moral philosophy. Additional backgrounds represented among core consultants include medicine, law, biological sciences, philosophy, social sciences, and public health/epidemiology. Only a few reported consultants with a background in nursing or the humanities. While respondents in 2010 indicated their faculty had similar academic backgrounds, many more reported having consultants with a background in humanities [3]. The majority of RECS include consultants with direct experience conducting empirical or clinical research (see Table 4). This type of expertise was not asked about in 2010. Only four RECS surveyed train and mentor fellows who participate in the delivery of consults

**Table 4.** Academic backgrounds of core consultants

Academic backgrounds represented	Background on Team (% of 42 RECS)
Bioethics/Applied Moral Philosophy	38 (90)
Medicine	24 (57)
Law	17 (40)
Biological sciences	17 (40)
Philosophy	16 (38)
Social Science	15 (36)
Epidemiology/Public Health	14 (33)
Nursing	3(17)
Humanities	3 (17)
**Direct research experience**	Experience on team (% of 42 RECS)
Empirical social science research	33 (79)
Clinical research	30 (71)
Basic laboratory research	19 (45)

RECS = research ethics consultation service

*Funding Sources:* RECS surveyed report that they are “financially supported” in three key ways (exclusively or in combination): external funds (including CTSA funds) and internal funds as part of academic service. One RECS offers its services for a fee (see Table 5). In 2010, three quarters of respondents reported their RECS being supported with CTSA funds and the majority indicated their RECS did not exist prior to the availability of CTSA funds.

**Table 5.** Funding source

Funding Source	n* (%)
External funding (e.g. NIH, CTSA, CIHR)	23 (55)
Internal funding	17 (40)
Fee-for-Service	1 (2)
Academic service (in kind)	12 (29)
Not sure	2 (5)

* Frequencies do not total 43 because respondents could pick more than one category.

NIH = National Institutes of Health

CTSA = Clinical and Translational Sciences Award

CIHT = Canadian Institutes for Health Research

*Processes:* While all RECS surveyed reported receiving requests through a variety of routes (e.g. website, email), the most common request mode is personal contact with a consultant (72%). More than two-thirds of the RECS surveyed (65%) reported logging and tracking consultation requests and almost all of those (96%) report doing so via an electronic database. Only half of RECS surveyed in 2010 had electronic tracking systems. Less than half of the RECS surveyed usually or always provide a written report to the requestor (40%). In 2010, only three RECS provided a written report to the requestor. Almost all RECS surveyed advertise their services on a website. RECS surveyed also make their services known by networking with investigators and by offering consultants educational sessions (see Table 6). Respondents in 2010 were not asked about advertising.

**Table 6.** Consult procedures

How consults are requested	RECS using mode (% of 42 RECS)
Personal contact with consultant	31 (74)
Central website	21 (50)
Central phone number	17 (41)
Central email	17 (41)
Page operator	4 (10)
**How service promoted**	
Website	37 (90)
Personal networking with researchers	28 (68)
Educational sessions initiated by consultants	22 (54)
Regular presence at investigator and Department meetings	8 (20)
Advertising with posters and fliers and in institutional newsletters	8 (20)

### Evaluation

About a quarter of RECS surveyed (28%) actively evaluate their services, primarily through satisfaction surveys routinely shared with requestors. In 2010, respondents were asked about whether their RECS had developed evaluation criteria: 1 in 10 stated they had evaluation criteria. The difference between 2010 and 2021 in terms of number of RECS conducting evaluation is not statistically significant (see Table 2). Only two RECS surveyed report evaluating the *outcomes* of their consults and do so by tracking successful IRB applications approved and grants secured after a consult.

Through an open-ended question, survey respondents were asked to imagine they have unlimited resources to evaluate the outcome of their consults (“If you had unlimited resources (human and financial) how would you evaluate the outcomes of your consults?”). The majority of the survey respondents focused on the consult itself as the object of their evaluation. The most common response was to obtain feedback from the requestor on their satisfaction with the consult (63%). A few respondents wanted to look at specific outcomes of each consult, including asking requestors if they felt their questions were answered (3) and whether recommendations made by the RECS were followed (4). A few also suggested that requestors should be asked to evaluate their experience after some time has passed (e.g. 6 months) (3). Four respondents suggested that a particular outcome of interest would be whether receipt of a research ethics consultation had an impact on the number of cycles of review conducted on a research proposal when a consult is obtained in advance of submission. Another small subset of respondents suggested ways to evaluate across consultations including descriptions of consults completed and the review of consults to identify potential gaps in knowledge of investigators and/or areas for policy development.

The remaining suggestions covered a range of stakeholders, including IRB/REBs, the research community, and institutional leadership, that could be queried about their awareness of, and/or satisfaction with, the RECS. Four respondents suggested a form of oversight by an independent group of experts. Finally, two respondents suggested a randomized trial be conducted to determine if research proposals provided with a research ethics consult in advance of their submission to the IRB are more likely to be approved than those that have not.

### Competencies

As noted above, the original competencies were developed and presented in a previous publication [10]. We organized the 27 competencies into four categories: knowledge (eight competencies), assessment (three competencies), process (nine competencies), and interpersonal (seven competencies) to elicit survey respondents’ opinions about the most important competencies a RECS should embody (i.e. across one or more consultant). Respondents were asked to select between one and three options per category. Table 1 displays all 27 competencies by category and in order from most to least common response by category. The eight competencies identified by at least half of those surveyed are bolded.

## Discussion

With the passing of 10 years, some general characteristics have changed and some have stayed the same. In 2021, we identified a larger number of RECS to survey and in turn received more responses. One consequence of the larger sample is the larger number of total consults completed in the last year although almost two-thirds of RECS completed fewer than 10 consults in the last year; less than 1 every month. Whether this volume is low, high, or just right is an important topic to explore further. There may be natural or artificial reasons that the supply and demand are or are not in sync. Of note, the number of RECS relying on a smaller number of consultants has increased. This could reflect an adjustment in supply to meet the demand. For instance, perhaps the number of investigators and study teams in need of consultation has decreased because they have all gained knowledge from past consults. On the other hand, perhaps the supply of consultants has led to limits in the number of consults that can be completed by a particular RECS. In addition, it points to the possibility that clinical ethics consultation services are being asked to take on research ethics topics [4]. Regardless better coordination between services offering clinical ethics and research ethics consults provided by two different groups of faculty and staff is warranted [13,14,15]. Another increase of note is that many more RECs in 2021 reported formally tracking their consults. There are multiple advantages to tracking consults including a way to track demand, tallying the types of consults completed, and an opportunity to tailor research ethics training on common topics of concern for a specific RECS. Another area ripe for additional data collection is source of funding. As noted, the 2010 sample of RECs was all affiliated with institutions that had CTSAs and almost all (97%) of funding came from external sources. The 2021 sample went beyond RECS affiliated with a CTSA and respondents were less likely to report external funding (55%). This difference could be explained by the shift in the sample but could also be related to the fact that subsequent CTSA requests for applications did not require that institutions commit to funding ethics initiatives as a component of their effort. In other words, many RECS surveyed in 2010 reported that they relied on CTSA funds to get started and have found other sources of funding to continue their service.

While the number of RECS reporting that they generate reports of their consults increased in the last decade, still only half reported always or usually doing so. Creating reports of consults is essential to advancing the field in terms of sharing experiences across RECS, evaluation of the content and process of analysis, and identifying and refining professional competencies.

### Evaluation

The number and proportion of RECS surveyed reporting that they evaluate their services more than doubled from 2010 to 2012 (most doing so via “customer” satisfaction surveys). A number of recent publications have brought attention to the importance of evaluating more than customer satisfaction [2,10]. Indeed, identifying mechanisms to rigorously evaluate RECS beyond customer satisfaction is one way for RECS to show evidence of the value they add to an institution’s research infrastructure. The 2021 respondents shared a range of ideas about approaches one could take more broadly to evaluate RECS. We believe at least three initiatives should be considered. First, additional work should be done to identify and measure substantive outcomes. For example, we have no empirical evidence that the availability of RECS contributes to the research ethics knowledge and ethical analysis skills of a research community. This hypothesis could be tested with a pre/post-test assessment of knowledge and skills, either coincident with the launch of a new RECS, or after an aggressive advertising of an existing RECS. However, while the findings might be valuable and interesting, such an evaluation would rely on a demonstrable increase in the current volume of consults at most RECS. A second approach would be to identify a set of key outcomes relevant to individual and institutional success and consider whether and how the availability of RECS may advance those outcomes. For example, the speed of IRB review and approval or number of grants funded. While it will be challenging to determine the role of the RECS compared with other related inputs tracking consults that are conducted in advance of IRB approval and/or during the grant writing process and following up with investigators about whether they felt the RECS played a role in their success could be a good starting point. A third approach is to systematically evaluate the narrative found in databases of research ethics consultations to better understand ethical issues that arise in the context of consultation services as well as the ethical analysis applied to resolved the issues identified [16]. Advancing this work is worthwhile in itself as well as capable of findings that could enhance any efforts to identify key competencies needed to provide high-quality consults.

### Competencies

A number of recent publications have focused on the need to consider the training and/or qualifications one must have to be a research ethics consultant [4,17,18]. Our goal in asking those actively engaged in consultation was to identify key competencies from the practice perspective. Future work can be directed at whether the content and categorization of the competencies are appropriate. For example, we placed three different competencies related to ethics in the knowledge category. These competencies may belong in their own category and/or need further definition to be more clearly distinguishable from each other. A consensus on competencies is key as the first generation of consultants will need to be replaced and more consultants trained [2]. Related to the idea of training is the identification of a set of core competencies that individual consultants and/or a group of individuals staffing a RECS ought to meet. This is based on the notion that a robust RECS needs a breadth of expertise in order to best serve its institution. Arnold and colleagues for example, present a list of 20 knowledge and skill-based competencies covering five key domains that guide the Medical University of South Carolina Clinical Research Ethics Fellowship program [17]. The list of competencies included in the 2021 survey came from a publication by Taylor and colleagues drawing on ASBH efforts to establish competencies for clinical ethics consultation [10]. While we (and others) do not believe that the field of research ethics is ready for a formal process of certification based on a comprehensive set of competencies, we do believe that such a list will allow RECS to consider their current and future needs when considering their ability to provide high-quality consultation today and tomorrow [4,19]. For example, questions such as whether to expand the scope of what RECS provide or whether smaller institutions ought to establish a RECS or collaborate with larger institutions that do can be addressed substantively and critically using the identified competencies as a guide [20,21].

## Limitations

While we believe we were able to identify the majority of RECS in the USA, our ability to identify all RECS in Canada was limited. An exploration of the who, what, where, and how of research ethics consultation in Canada is warranted. In 2010, the survey of RECS was sent only to RECS affiliated with a CTSA [3]. It is possible that there were additional services in 2010 that were not part of the sampling frame at that time, but our knowledge of the landscape of RECS leads us to believe there has been an increase in the absolute number of RECS in the USA.

## Conclusion

The findings from our survey demonstrate that there have been growth and development of RECS since 2010. Further developing evaluation and competency guidelines will help existing RECS continue to grow and facilitate newly established RECS maturation. Both will allow RECS personnel to better serve their institutions and add value to the research conducted.
